# The Distribution of Minerals in Crucial Plant Parts of Various Elderberry (*Sambucus* spp.) Interspecific Hybrids

**DOI:** 10.3390/plants10040653

**Published:** 2021-03-30

**Authors:** Nataša Imenšek, Vilma Sem, Mitja Kolar, Anton Ivančič, Janja Kristl

**Affiliations:** 1 Faculty of Agriculture and Life Sciences, University of Maribor, Pivola 10, 2311 Hoče, Slovenia; vilma.sem@um.si (V.S.); anton.ivancic@guest.um.si (A.I.); janja.kristl@um.si (J.K.); 2 Faculty of Chemistry and Chemical Technology, University of Ljubljana, Večna Pot 113, 1000 Ljubljana, Slovenia; Mitja.Kolar@fkkt.uni-lj.si

**Keywords:** interspecific hybrids, minerals, plant parts

## Abstract

In view of growing requirements of the food industry regarding elderberries (genus *Sambucus*), a need to increase their productivity and improve their chemical composition has emerged. With this purpose in mind, numerous elderberry interspecific hybrids have been created. In the present work, the content of minerals in their crucial plant parts was studied. It was also investigated whether superior genotypes regarding the mineral composition of berries and inflorescences could be predicted at early stages of plant development. The results showed that elderberry leaves contained the highest amounts of Ca, Mg, Mn, Zn, and Sr, while K and P were predominant in fruit stalks. Fe and Al prevailed in roots and Cu in bark. Although berries showed lower mineral content compared to other plant parts, their mineral content is not negligible and could be comparable to other commonly consumed berries. Genotypes with a favorable mineral content of inflorescences and berries could be predicted on the basis of known mineral composition of their shoots and leaves. The study also indicates that *S. nigra* genotypes and the majority of interspecific hybrids analyzed are suitable for further genetic breeding or cultivation.

## 1. Introduction

Both humans and plants require a sufficient intake of individual essential minerals, although requirements for some minerals is greater (macroelements) than for others (microelements) [1,2] and varies between individual organisms and species. In the human body, the presence of minerals is needed to form various body structures and regulate certain metabolic and physicochemical processes that are crucial for life [3]. Some macro and microelements are components of bones (Ca, Mg, Mn, P, B, and F) and teeth (Ca, P and F), while most microelements (Cu, Fe, Mn, Se, and Zn) play a key role as a structural, catalytic, or binding factor in many enzymatic processes. K, Ca, Mg, P, and Na have significant functions in transmission and signaling in neurons, and they are also important inorganic electrolytes involved in ionic and osmotic balance and electrical gradients, together with Cl, and S [4,5]. The mineral elements most often lacking in population diets are Fe, Zn, Cu, Ca, Mg, I, and Se. From available data, it is estimated that about 60% of the world’s population is affected by Fe deficiency and 15% by Se deficiency [6]. Compared to animal products, plants represent a very important source of minerals for humans [4], as they contain almost all minerals considered as essential for human nutrition [7]. However, for plants, each of the minerals has important roles in several biochemical processes. Similar to for humans, Ca plays a role as a structural component in the cell wall and membranes of plants, enabling intracellular exchange of molecules. Some macroelements (N, S, P) have an important role as building blocks of nucleic acids and proteins. K and Cl have a large role in the osmotic potential of cells and tissues of glycopyhtic plants and stabilize the optimal pH for most enzyme reactions. Mg and micronutrients (except Cl) serve predominantly as components of enzyme molecules, although the main function of Mg is being the central atom of the chlorophyll molecule. Deficiency of any vital mineral can seriously inhibit plant growth and development [1].

The content of minerals in plants depends on numerous factors, such as the type and chemical composition of the soil, soil fertility, the root-soil interface, the characteristics of the absorption mechanism, and translocation within the plant [8]. Translocation begins in the soil, where minerals cross the root via apoplastic and/or symplastic pathways to the stele and are loaded into the xylem for transport to the transpiring leaf tissues (shoots and leaves). The recirculation of minerals within a plant and their translocation to a specific part of the plant where they are needed (usually from an older to a younger tissue) occurs through the phloem. Some minerals are readily transported (Mg, K, P, and S), some are less mobile (Fe, Zn, Cu, and B), and others (Mn and Ca) are essentially immobile in the phloem of most plant species. However, minerals with low mobility in the phloem can also be transported through the xylem [9]. The mineral content of individual plant parts is highly dependent on the translocation of minerals, which varies according to plant species, plant tissue, growth stage, and environmental conditions [10].

Among plant species, especially among common fruit and vegetable plants, the black elderberry (*Sambucus nigra*) is considered as an important source of nutrition, characterized by high color capacity, antioxidant activity [11,12,13,14,15,16,17,18,19,20], and relatively large amounts of minerals [21,22]. According to the taxonomy of Bolli [23], *S. nigra* represents one of the nine species of the genus *Sambucus* which belongs to the family Adoxaceae [24]. They grow as small trees, shrubs, of various forms or herbs and are native to sun-exposed sites almost all over the world [25,26]. They are known to be among the oldest cultivated plants [27], having been used for nutritional and medicinal purposes in prehistoric times [28,29]. Nowadays, there is an increasing interest in elderberry plants, especially due to their wide distribution and availability, ease of cultivation, favorable chemical composition [30], and usability of almost all major plant parts (roots, bark, leaves, shoots, inflorescences, and berries) [31,32,33].

Many authors studied the chemical composition of elderberry; however, their research was focused mainly on the content of some organic substances (phenols, sugars, organic acids, and vitamin C) in fruits (berries) and inflorescences of the European (*S. nigra* subsp. *nigra*) [21,25,27,34,35,36,37,38,39,40,41,42] and the American black elderberry (*S. nigra* subsp. *canadensis* (L.) R. Bolli) [24,43,44,45,46]. The findings of these authors are similar and mostly describe fruits and inflorescences as a significant source of phenolic compounds and vitamin C with a favorable ratio between sugars and organic acids. According to some other authors, elderberry fruits are also an important source of some minerals [47]. They are superior in K, Mg, and P content compared to some Madeiran blueberry and blackberry cultivars [48] and are very high in K and Mg content compared to some herbs (yellow bedstraw, thyme, yarrow, and wild garlic) [28].

There is a scarcity of data on the chemical composition of individual elderberry plant parts, as only data on the content of phenolic compounds in leaves and inflorescences are available [37,49,50]. To our knowledge, there are no data on the mineral content in other plant parts (i.e., fruit stalks, leaves, shoots, bark, and roots) of different elderberry species or their interspecific hybrids. Since minerals are among the crucial nutritional components and play an important role in plant [1] and human [5] metabolism, the main purpose of this study was to analyze the mineral content of inflorescences and fruits, the most commonly used plant parts of elderberry. To improve the knowledge about the mineral composition of the whole elderberry plant, minerals were also determined in fruit stalks, leaves, shoots, bark, and roots. In addition, it was investigated whether genotypes with a favorable mineral composition of berries and inflorescences could be predicted from the known mineral composition of their plant parts present earlier in the season or in earlier developmental stages such as shoots and leaves. The results also enable the identification of the most promising genetic combinations (genotypes) that could be used in further cultivation and breeding processes.

## 2. Results and Discussion

### 2.1. The Content and Distribution of Macronutrients in Various Plant Parts of Elderberry Interspecific Hybrids

**Figure 1:**
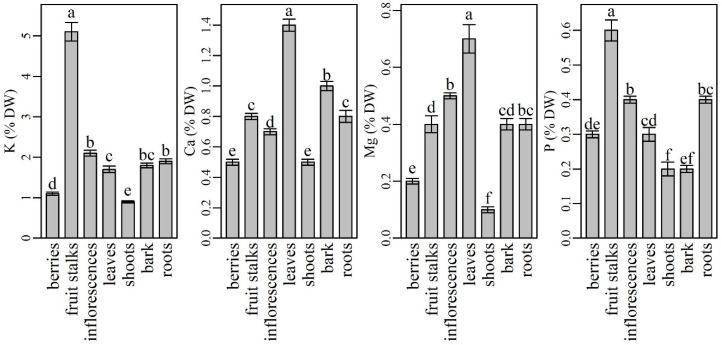
The content of macroelements in different parts of elderberry plants. Means labelled with the same letter are not significantly different (Tukey, *p* ≤ 0.05).

Among the macronutrients, potassium (K) was analyzed in the highest amounts in all plant parts (Figure 1). However, shoots contained significantly lower (0.86 ± 0.03%) and fruit stalks significantly higher K levels (5.09 ± 0.23%) than other plant parts. Inflorescences were found to be the second richest plant part in K, followed by similar K levels in roots and bark and significantly different K levels in leaves and berries. Our results are partly in agreement with data from the literature. Namely, K is known as a highly phloem mobile element that is usually translocated from older to younger tissues [51]. According to Tagliavini et al. [52], fruits and fruit stalks are its major sink. However, similar to our results, Twyford and Walmsley [53] noted that fruits stalks and inflorescences were the most richest tissues with K in the fruiting phase of the banana tree. When comparing K contents in the most frequently used elderberry plant parts (inflorescences and berries), our results agree with those of Kolodziej et al. [54], who described elderberry inflorescences as richer in K than in berries, although their reported values were higher than ours. K is known for its beneficial effects in protecting humans from cardiovascular disease, thus the inclusion of K-rich foods in the human diet has recently been increasingly recommended [55]. According to our findings, elderberry fruit stalks, inflorescences and roots could represent a good source of K in human diet. However, the use of fruit stalks and roots cannot be recommended due to their potential oral toxicity [56] and difficult accessibility. Although berries have low K content compared to other parts of the plant, they are still a better K source than some other berries (blackberry, blueberry) [48] and herbs [28].

Calcium (Ca) was the second most abundant mineral determined in the elderberry plant. Among the plant parts analyzed, leaves had the highest Ca content (1.38 ± 0.04%), followed by bark (0.96 ± 0.03%), fruit stalks (0.84 ± 0.02%), roots (0.81 ± 0.04%), and inflorescences (0.68 ± 0.02%). All the values (except those for fruit stalks and roots) were significantly different. Compared to other plant parts, the significantly lowest Ca content was found in berries and shoots (0.54 ± 0.02% and 0.54 ± 0.02%). Our results are in agreement with Tagliavini et al. [52], who studied the mineral content in strawberry plants. They found the highest Ca content in leaves, followed by fruits, fruit stalks, and roots. Our results are also in agreement with some other authors who describe Ca as a mineral that is poorly mobile from leaves to phloem-fed tissues with low transpiration rates such as fruits and flowers [1,57,58]. In fruits, Ca is accumulated at the beginning of fruit growth via the xylem, which in some fruits becomes increasingly dysfunctional as fruit development progresses [59]. Consequently, fruits usually have lower Ca concentrations at the end of season compared to leaves. On the other hand, the growing part of the leaf tissue requires higher Ca concentrations, which accumulate and remain in leaves [57] because Ca is immobile through the phloem. When comparing Ca levels in berries and inflorescences, our results are in accordance with those of Młynarczyk et al. and Kolodziej et al. [39,54]. Authors found that inflorescences were richer in Ca than berries. However, despite a lower Ca content in comparison to inflorescences, berries were reported to be a rich source of Ca [60]. According to our results, the consumption of 100 g of berries could cover 13% of the recommended daily intake of Ca for women and men.

Among the plant parts analyzed, leaves had significantly higher magnesium (Mg) contents (0.73 ± 0.05) and shoots (0.15 ± 0.01) had significantly lower Mg contents than other plant parts. Inflorescences were the second richest source of Mg followed by roots, fruits stalks, and bark. Berries contained significantly less Mg than inflorescences. The results from some previous studies are inconsistent. Our results agree with those reported by Kolodziej et al. [54], while Młynarczyk et al. [39] described elderberry fruits as several times richer in Mg than inflorescences. Although elderberry fruits turned out to be among the poorest plant parts in Mg, according to Imbrea et al. and Młynarczyk et al. [28,48], they are richer in Mg than some blueberry and blackberry cultivars and some herbs. With the respect to other plant parts, our results are in agreement with Wilkinson et al. [61], who stated that Mg is a highly phloem-mobile element that is readily translocated to reproductive organs such as fruits, seeds, and tubers, which have the first priority on Mg supply. Subsequently, when Mg supply in these organs approaches adequacy, vegetative structures (stem, including bark, leaves, and roots) become storage sinks for Mg. Moreover, our results regarding the highest Mg levels in leaves are also in agreement with those reported by Karley and White [62], who found that most Mg in plants is bound or incorporated in cellular compartments, with the highest concentrations in chloroplasts, i.e., leaves. As Rosanoff et al. [63] documented, inadequate intake and low nutritional status of Mg occurs in many populations worldwide, therefore preparations and products from elderberry leaves and inflorescences could be a good source of Mg in the human diet.

In elderberry plant, phosphorus (P) was determined to have the highest amounts in fruit stalks (0.60 ± 0.03%), followed by significantly lower contents in the inflorescences (0.45 ± 0.01%) and roots (0.36 ± 0.01%). Leaves (0.30 ± 0.02%) and berries (0.26 ± 0.01%) contained even less P, but their contents were not significantly different from each other. Bark and shoots contained the lowest amounts of P (0.23 ± 0.01%, 0.21 ± 0.02%, respectively). The observed results are partly in agreement with the data reported by other authors. After absorption, P is loaded from the roots into the xylem and distributed further into the shoots. Then, it accumulates in the leaves during the plant growth phase until leaf senescence. Thereafter, plants remobilize P from the senescing leaves into the reproductive structures, especially the seeds [64]. This could partly explain the high P contents in elderberry inflorescences and fruits stalks, as this is the beginning of P translocation to seeds. However, high P levels are not necessarily desirable, especially when most of the P is in the form of phytate (e.g., in cereal grains and legume seeds), which is not absorbed by humans and limits the bioavailability of dietary iron and zinc [6]. According to Marschner [1], P deficiency is rare in the human diet, so the P intake is below the estimated average requirement for only 5% of the adult population.

### 2.2. The Content and Distribution of Micronutrients, Aluminium and Strontium in Various Plant Parts of Elderberry Interspecific Hybrids

**Figure 2:**
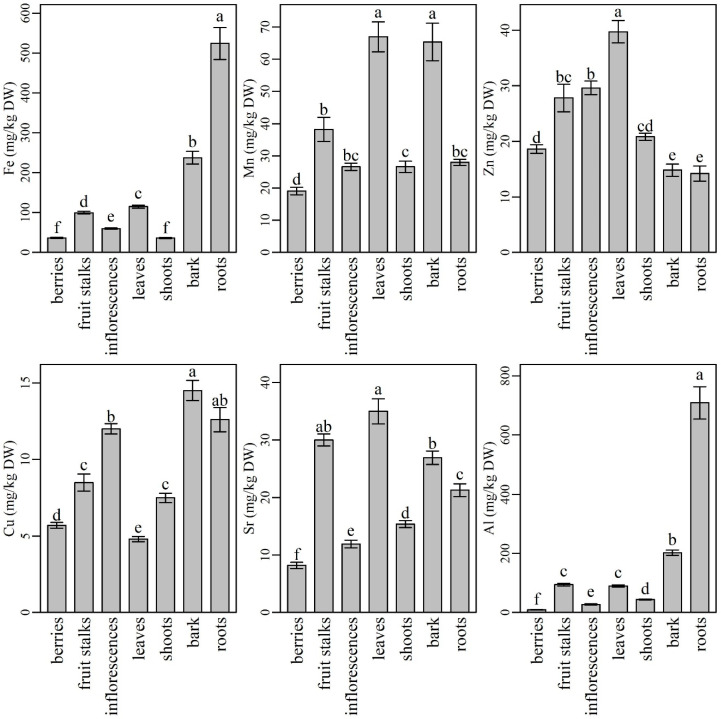
The content of microelements, Sr, and Al in different parts of elderberry plants. Means labelled with the same letter are not significantly different (Tukey, *p* ≤ 0.05).

Among micronutrients, iron (Fe) was analyzed in the highest amounts in all plant parts (Figure 2). Its content was the highest in roots (524 ± 53 mg/kg DW), followed by significantly lower levels in bark (237 ± 16 mg/kg DW), leaves (115 ± 4 mg/kg DW), fruit stalks (99.5 ± 3.4 mg/kg DW), and inflorescences (59.7 ± 1.8 mg/kg DW). The lowest Fe contents were determined in berries (36.4 ± 1.4 mg/kg DW) and shoots (36.0 ± 1.4 mg/kg DW). Our results are in accordance with those reported by Page and Feller [65], who found that Fe is retained in roots in some cases due to insolubility or cell compartmentalization, which prevents its delivery to the xylem. However, Fe entering the xylem is usually retained in older leaves because of its poor phloem mobility. Similarly to our results, the lowest Fe content in plant shoots and fruits was also found in tomato plants by Singh et al. [66]. Inflorescences turned out richer in Fe as berries. This finding is in agreement with the results reported by Młynarczyk et al. [39], who also compared the mineral content of elderberry fruits and inflorescences. Our results indicate that berries are among the plant parts poorest in Fe; however, when compared to blueberries, raspberries, and cranberries, they contain similar or even higher Fe levels [67]. Since Fe deficiency is the most common and widespread nutritional disorder in the world [1], the use of elderberry leaves, inflorescences, and fruits could positively contribute to Fe intake. Despite the high Fe content, the use of roots, bark, and fruits stalks could not be recommended [56].

The second most abundant microelement in the elderberry plant was manganese (Mn). Compared to other plant parts, its content was significantly higher in leaves (67.0 ± 4.6 mg/kg DW) and bark (65.4 ± 5.8 mg/kg DW), followed by fruit stalks (38.2 ± 3.7 mg/kg DW), roots, shoots, and inflorescences. Berries were characterized with significantly lower Mn content (19.1 ± 1.2 mg/kg DW) compared to the other plant parts. Our results are in agreement with Marschner [1], who documented that Mn is characterized by only a minor redistribution within the plant and accumulates primarily in the photosynthetically active (transpiring) leaves. Our results are also in agreement with those reported by Młynarczyk et al. [39], who described elderberry inflorescences as richer in Mn than berries and documented similar Mn levels to the ones we did. Our results are also similar to the results of Diviš et al. [47], who considered elderberry fruits as an important source of some microelements including Mn. According to our results, the use of elderberry leaves could contribute to higher Mn intake, while the bark, despite its relatively high Mn content, could not be recommended for use because of its difficult accessibility and also because harvesting the bark is destructive for the plant.

Zinc (Zn) predominated in elderberry leaves (39.7 ± 2.0 mg/kg DW), followed by significantly lower levels in inflorescences and fruit stalks. In contrast, the lowest Zn contents were found in bark (14.8 ± 1.1 mg/kg DW) and roots (14.2 ± 1.8 mg/kg DW), while shoots and berries turned out as medium rich Zn supply with non-significant differences in Zn levels. Zn has good mobility and is transported to growing plant parts through the phloem [68]; therefore, its content in plant roots and bark generally does not exceed its content in leaves [69]. Adequate Zn intake is considered to be essential for the proper activity of a number of enzymes. In recent years, symptoms of Zn deficiency have been observed in some human populations, especially in those consuming diets high in phytate and low in meat [1]. Consequently, the demand for Zn-rich dietary supplements or foods has greatly increased. According to our findings, elderberry leaves, inflorescences, and berries, or their products, could be important sources of Zn in the human diet.

The highest Cu content was found in elderberry bark (14.5 ± 0.7 mg/kg DW). Elderberry roots contained slightly less Cu than bark, but their contents did not differ significantly. Inflorescences contained similar Cu level to roots, while fruit stalks and shoots had significantly lower Cu levels compared to bark, roots, and inflorescences. Significantly lower Cu contents were determined in elderberry fruits (5.70 ± 0.19 mg/kg DW) and leaves (4.83 ± 0.16 mg/kg DW) when compared to other plant parts. Our results are in partial agreement with authors who have studied the metal content in other plants such as some wetland plants [70,71,72] and metallophyte species [73]. The authors found that plant roots were the richest in Cu compared to plant shoots and leaves. On the other hand, their results were different when they compared Cu content in plant roots and stem. In most cases, the roots contained higher Cu amounts than the stem, but in the case of Indian sage (*Pluchea indica*), the authors’ results were similar to ours, where part of the elderberry stem (bark) was richer in Cu than the roots. Among the most frequently used elderberry plant parts, inflorescences proved to be richer in Cu than in berries. Similar results and Cu levels considering elderberry inflorescences and fruits were obtained by Młynarczyk et al., [39]. Since Cu is associated with numerous enzyme systems in human metabolism [1], its sufficient intake is very important. For this reason, the inclusion of elderberry inflorescences in the human diet could be suggested.

The highest Sr contents were found in leaves (35.0 ± 2.2 mg/kg DW) and fruit stalks (30.0 ± 1.0 mg/kg DW). Bark contained lower Sr levels than fruit stalks, but their levels were not significantly different, while Sr levels in roots were significantly lower compared to bark. Shoots, inflorescences, and berries were characterized by the lowest Sr contents, all significantly different from each other (15.4 ± 0.6 mg/kg DW, 11.9 ± 0.7 mg/kg DW, and 8.17 ± 0.55 mg/kg DW, respectively). The increased Sr content in elderberry leaves was most likely caused by its movement from roots to the stem (including bark) and further to the newly formed growing leaves (shoots). Thereafter, most of the Sr accumulated in leaves and probably in other green parts (green stalks). Only small amounts of Sr were transported from leaves to inflorescences and even less to berries. Moreover, according to our results, the second highest Sr level accumulated and remained in fruit stalks. These results are in agreement with those reported by Gouthu et al. [74], who studied Sr translocation in soybean and found the highest Sr contents in plant leaves and the lowest in flowers and fruits. Since Sr is a poorly phloem-mobile element that is distributed in plants mainly though the xylem [1], its contents in flowers and fruits are low. Most likely, Sr also accumulated slowly in the green stalks and remained there at the time of plant maturation. In the human body, smaller quantities of Sr could have positive affect in metabolic bone diseases [75]. On the other hand, when overdosed, Sr could have various toxic effects on lungs and the reproductive system [76]. Since elderberry inflorescences and berries contained lower levels of Sr than some more frequently consumed foods such as leafy greens, grains, and seafood [77], they could still be safely used in human nutrition.

Elderberry roots showed the highest Al content (709 ± 73 mg/kg DW), followed by significantly lower contents in bark, fruit stalks, and leaves. Inflorescences and berries showed the lowest Al contents (27.4 ± 1.9 mg/kg DW and 9.37 ± 0.61 mg/kg DW, respectively). Since Al is not mobile between leaves and cannot be transported through the phloem [78], its low levels in plant shoots, inflorescences, and fruits are consistent with the available data from the literature. Moreover, according to Marschner [1], Al is retained in the roots of some plant species and, in general, its content in plant tops is much lower than in roots [79]. Since Al is a toxic element to humans [80], its low content in the most commonly used plant parts is highly desirable. Elderberry fruits and inflorescences could be recommended for further use in the food industry, while leaves should be less included in the human diet.

### 2.3. Clustering of Elderberry Interspecific Hybrids and Correlation for Minerals between Plant Parts

All genotypes analyzed were clustered into four groups. The characteristics of each group (high or low mineral content of their inflorescences and berries) are presented in Table 1, Supplemental Material (Figures S1–S3). Compared to other genotypes analyzed, the inflorescences and berries of ((JA × NI) × NI) × ((JA × NI) × BB), ((JA × NI) × RAC) × ((JA × NI) × BB), and (JA × (JA × MIQ)) × ((JA × NI) × BB) were characterized with the poorest mineral composition, followed by JA × CER No 3 C1 and ((JA × NI) × SIB) × CER, which on the other hand also contained high Al levels in the berries. These genotypes should be less used or avoided in further breeding or consumer useage. Similar but more preferable results were obtained for *S. nigra* (NI) and its varieties *S. nigra* var. *viridis* (VIR), *S. nigra* var. *laciniata* (LAC), and *S. nigra* ‘Black Beauty’ (BB). These genotypes were superior with respect to some minerals in berries (Ca and Zn) and inflorescences (Ca, Zn, Mg and Fe), but contained higher levels of Sr (berries and inflorescences) and Al (inflorescences) compared to the previously mentioned genotypes. However, the levels of undesirable Sr and Al in these genotypes did not exceed their average levels in other berry fruits such as cranberries, lingonberries, and blueberries [81]. Genotypes from group 2 (Table 2) showed the most desirable mineral composition and could therefore be recommended among *S. nigra* and its varieties for further inclusion in breeding processes or use in the food industry.

Genotypes with favorable mineral composition of inflorescences and berries could be predicted partly on the basis of the known mineral composition of their shoots and leaves. Indeed, the majority of correlations for mineral content between the above mentioned elderberry plant parts were significant and positive (Table 2). In general, the correlations for mineral content between shoots and inflorescences or berries were weaker than the correlations for mineral content between leaves and inflorescences or berries. The results obtained showed that the genotype with higher K, Mg, Fe, Cu, Mn, and Sr content in shoots or leaves was also, in general, characterized by higher K, Mg, Fe, Cu, Mn, and Sr content in inflorescences. In addition, the genotype with higher K, Ca, Cu, Mn, Zn, and Sr content in shoots or leaves was also characterized with higher K, Ca, Cu, Mn, Zn, and Sr content in berries. With regard to inflorescences and berries, the results obtained showed that the correlations between mentioned plant parts were positive and significant for most minerals. The exceptions included the correlation for Fe content, which was not significant, and the correlation for Al content, which was negative and significant. In general, the genotype with higher K, Ca, Mg, P, Cu, Mn, Sr, and Zn content in inflorescences showed higher content of these minerals in berries. In contrast, the genotype with higher Al content in inflorescences was characterized with lower Al content in berries.

## 3. Materials and Methods

### 3.1. Plant Material—Elderberry Genotypes and Samples

The plant material for this study included 47 elderberry genotypes, of which five belonged to the species *Sambucus nigra* (two local genotypes belonging to *S. nigra* subsp. *nigra* according to Bolli, 1994 [23], *S. nigra* var. *viridis*, *S. nigra* var. *laciniata*, and the cultivar *S. nigra* ‘Black Beauty’), and the rest were interspecific hybrids (Table 3). All interspecific hybrids included in this study involved a self-incompatible genotype *S. javanica* (Chinese or Javanese elderberry) originating from the Island of Espiritu Santo, Vanuatu. Since the parental material of interspecific hybrids was assumed to be highly heterozygous, each offspring individual originating from the same cross represented a different genotype. Most of hybrids belonged to the third cycle of the recurrent selection program and were created at the University of Maribor, Faculty of Agriculture and Life Sciences at Hoče near Maribor, Slovenia. The majority of analyzed plants originated directly from seed. The exceptions were two C1 clones (*S. javanica* × *S. nigra*) × *S. nigra* ‘Black Beauty’ and *S. javanica* × *S. cerulea* hybrid No 3. The sampled plants were three to four years old shrubs. Berries, fruits stalks, inflorescences, leaves, shoots, bark, and roots were taken from each plant studied. Since shoots were first available, they were also sampled first. Afterwards, other plant parts were taken at the same date (when they were all available). Since each genotype matured at different dates, plants were sampled over a range of dates. After sampling, the plant material was frozen in liquid nitrogen, freeze-dried (Christ Alpha 1-2 LD; Vacuumbrand GMBH, Germany), crushed into a fine powder, vacuum packed, and stored at −80 °C until analyzed.

### 3.2. Chemical Analysis

For the determination of minerals, approximately 100 mg of sample was weighed into a long-necked Kjeldahl flask and 2 mL of conc. HNO_3_ and 1 mL of conc. H_2_SO_4_ were added. The samples were digested with heating of the mixture over a burner flame until no gas was emitted. After that, 1 mL of 30% H_2_O_2_ was added to the cooled mixture and the mixture was heated again until it became clear. The clear liquid was then diluted to 50 mL with 1% HNO_3_. Using multi-point calibration curves, typical at 10 µg/L, 30 µg/L, 100 µg/L, 300 µg/L, and 1000 µg/L, with the Varian AX Vista ICP/OES instrument, the metal (K, Ca, Mg, Fe, Mn, Zn, Cu, Sr and Al) content in the solutions was then determined. All analyses were performed in duplicates.

The content of P was determined according to a vanadate–molybdate method [82]. The absorbance was measured at 406 nm using Varian Cary UV-Vis spectrophotometer. The contents of minerals were expressed as mean ± SE (% DW for K, Ca, Mg, P and mg/kg DW for Fe, Mn, Zn, Cu, Sr, and Al).

### 3.3. Statistical Analysis

The contents of minerals among different plant parts were modeled by linear mixed models (LMER) with a fixed factor plant part and random factors genotype and plant. To estimate paired differences between plant parts, the Tukey multiple comparison test was applied. Spearman correlation coefficients were computed to gain better insight into the mineral distribution among plant parts.

Similarity of plants/genotypes considering mineral contents in different plant parts was studied by agglomerative hierarchical clustering (AGNES) with Euclidian distance metric and Ward’s linkage method. The inspection of dendrogram revealed that splitting our data into four clusters was appropriate. For further investigation of the properties of each cluster, principal component analysis (PCA) was implemented. To avoid redundancy in the presentation and interpretation of results, PCA was applied only to mineral contents in the nutritionally most important plant parts (berries and inflorescences).

The statistical analysis was performed using the statistical program package R [83] and libraries lme4 [84], lmerTest [85], cluster [86], and FactoMineR [87].

## 4. Conclusions

Among the elderberry plant parts analyzed, fruit stalks contained the highest content of K and P, followed by the inflorescences and leaves. Ca, Mg, Mn, Zn, and Sr predominated in the leaves, while Fe and Al dominated in the roots. The highest Cu levels were determined in the elderberry bark. Berries showed lower mineral content compared to the inflorescences and some other plant parts studied. When berries are used together with the fruit stalks, they have richer mineral composition than the inflorescences. However, the use of berries together with fruit stalks cannot be recommended due to the high Sr and Al content in fruit stalks, their potential toxicity and probably undesired impact on taste. Among the plant parts studied, shoots showed the lowest content of K, Ca, Mg, P, Fe, and Cu.

Regarding genotypes studied, *S. nigra* with its varieties and the majority of the interspecific hybrids analyzed (with exception of genotypes from group 3 and group 4) (Table 2) could be recommended for further breeding processes or direct consumers’ use, as they show diverse and rich mineral composition.

Genotypes with a preferential mineral composition of berries and inflorescences could be predicted partly based on known mineral composition of their shoots and leaves that are available earlier in the growing season or at earlier developmental stages. In addition, genotypes with better mineral composition of berries could also be predicted based on the known mineral composition of their inflorescences. The determination of genotypes with superior mineral composition of berries and/or inflorescences in advance could accelerate genetic breeding processes and contribute to the use of improved raw materials in the food industry.

## Figures and Tables

**Table 1:** Cluster analysis results with properties of inflorescences and berries of each cluster obtained from principal component analysis (PCA) analysis.

Clusters		Group 1	Group 2	Group 3	Group 4
		NI VIR LAC BB	JA × VIR (JA × NI) × BB C1 (JA × NI) × BB ((JA × NI) × BB) × (JA × (JA × NI)) JA × (JA × EB) JA × (((JA × NI) × NI) × MIQ) ((JA × NI) × BB) × (JA × MIQ) ((JA × NI) × SIB) × (JA × NI) JA × (JA × CER) ((CER × NI) × JA) × (CER × NI) (JA × RAC) × (CER × NI) ((JA × NI) × MIQ) × (CER × MIQ) ((JA × CER) × MIQ) × ((JA × NI) × CER) ((JA × NI) × SIB) × (JA × CER) No 3	((JA × NI) × NI) × ((JA × NI) × BB) ((JA × NI) × RAC) × ((JA × NI) × BB) (JA × (JA × MIQ)) × ((JA × NI) × BB)	JA × CER No 3 C1 ((JA × NI) × SIB) × CER
**Mineral content**	**High**	Ca (inflorescences) Ca (berries) Mg (inflorescences) Fe (inflorescences) Zn (inflorescences) Zn (berries) Sr (berries) Sr (inflorescences) Al (inflorescences)	K (inflorescences) K (berries) Mg (berries) Fe (berries) Mn (inflorescences) Mn (berries) Cu (inflorescences) Cu (berries)	K (inflorescences) K (berries)	Ca (inflorescences) Ca (berries) Zn (inflorescences) Zn (berries) Sr (inflorescences) Sr (berries) Al (berries)
	**Low**	K (berries) K (inflorescences) Mn (berries) Al (berries)		Ca (inflorescences) Mg (berries) Fe (berries) Mn (berries) Mn (inflorescences) Cu (berries) Cu (inflorescences) Zn (berries) Sr (berries) Sr (inflorescences)	K (berries) K (inflorescences) Mg (inflorescences) Fe (inflorescences) Mn (berries) Mn (inflorescences) Al (inflorescences)

**Table 2:** Correlation coefficients for mineral contents between different plant parts of elderberry interspecific hybrids.

**K**	shoots	leaves	Berries	inflorescences	**Cu**	shoots	leaves	berries	Inflorescences
shoots	1.00	0.61 ***	0.44 **	0.39 **	shoots	1.00	0.31 *	0.54 ***	0.46 **
leaves	0.61 ***	1.00	0.71 ***	0.62 ***	leaves	0.31 *	1.00	0.40 **	0.40 **
berries	0.44 **	0.71 ***	1.00	0.57 ***	berries	0.54 ***	0.40 **	1.00	0.60 ***
inflorescences	0.39 **	0.62 ***	0.57 ***	1.00	inflorescences	0.46 **	0.40 **	0.60 ***	1.00
**Ca**	shoots	leaves	Berries	inflorescences	**Mn**	shoots	leaves	berries	inflorescences
shoots	1.00	0.47 ***	0.41 **	0.23	shoots	1.00	0.83 ***	0.85 ***	0.68 ***
leaves	0.47 ***	1.00	0.69 ***	0.42 **	leaves	0.83 ***	1.00	0.77 ***	0.83 ***
berries	0.41 **	0.69 ***	1.00	0.50 ***	berries	0.85 ***	0.77 ***	1.00	0.69 ***
inflorescences	0.23	0.42 **	0.50 ***	1.00	inflorescences	0.68 ***	0.83 ***	0.69 ***	1.00
**Mg**	shoots	leaves	Berries	inflorescences	**Sr**	shoots	leaves	berries	inflorescences
shoots	1.00	0.54 ***	0.25	0.45 **	shoots	1.00	0.76 ***	0.77 ***	0.71 ***
leaves	0.54 ***	1.00	0.41 **	0.62 ***	leaves	0.76 ***	1.00	0.78 ***	0.73 ***
berries	0.25	0.41 **	1.00	0.48 ***	berries	0.77 ***	0.78 ***	1.00	0.78 ***
inflorescences	0.45 **	0.62 ***	0.48 ***	1.00	inflorescences	0.71 ***	0.73 ***	0.78 ***	1.00
**P**	shoots	leaves	Berries	inflorescences	**Zn**	shoots	leaves	berries	inflorescences
shoots	1.00	0.36 *	−0.22	−0.14	shoots	1.00	0.42 **	0.51 ***	0.45 **
leaves	0.36 *	1.00	0.01	0.47 ***	leaves	0.42 **	1.00	0.38 **	0.22
berries	−0.22	0.01	1.00	0.38 **	berries	0.51 ***	0.38 **	1.00	0.51 ***
inflorescences	−0.14	0.47 ***	0.38 **	1.00	inflorescences	0.45 **	0.22	0.51 ***	1.00
**Fe**	shoots	leaves	Berries	inflorescences	**Al**	shoots	leaves	berries	inflorescences
shoots	1.00	0.15	0.03	0.37 *	shoots	1.00	0.15	0.00	0.22
leaves	0.15	1.00	0.11	0.47 ***	leaves	0.15	1.00	−0.04	0.30 *
berries	0.03	0.11	1.00	0.27	berries	0.00	−0.04	1.00	−0.59 ***
inflorescences	0.37 *	0.47 ***	0.27	1.00	inflorescences	0.22	0.30 *	−0.59 ***	1.00

* sig. < 0.05, ** sig. < 0.01, *** sig. < 0.001.

**Table 3:** Elderberry species and interspecific hybrids included in the investigation.

Material	Abbreviation	No.
Species and other taxons		
*Sambucus nigra*	NI	2
*S. nigra* var. *viridis*	VIR	1
*S. nigra* var. *laciniata*	LAC	1
*S. nigra* ‘Black Beauty’	BB	1
Interspecific hybrids exhibiting combination of traits of *S. nigra* and *S. javanica*		
*S. javanica* × *S. nigra* var. *viridis*	JA × VIR	1
(*S. javanica* × *S. nigra*) × *S. nigra* ‘Black Beauty’C1 ^a^	(JA × NI) × BB C1	5
(*S. javanica* × *S. nigra*) × *S. nigra* ‘Black Beauty’ ^b^	(JA × NI) × BB	2
((*S. javanica* × *S. nigra*) × *S. nigra*) × ((*S. javanica* × *S. nigra*) × *S. nigra* ‘Black Beauty’)	((JA × NI) × NI) × ((JA × NI) × BB)	6
((*S. javanica* × *S. nigra*) × *S nigra* ‘Black Beauty’) × (*S. javanica* × (*S. javanica* × *S. nigra*))	((JA × NI) × BB) × (JA × (JA × NI))	1
Interspecific hybrids exhibiting similarity to *S. ebulus*		
*S. javanica* × (*S. javanica* × *S. ebulus*)	JA × (JA × EB)	1
Interspecific hybrids exhibiting similarity to *S. racemosa*		
((*S. javanica* × *S. nigra*) × *S. racemosa*) × ((*S. javanica* × *S. nigra*) × *S. nigra* ‘Black Beauty’)	((JA × NI) × RAC) × ((JA × NI) × BB)	2
*S. javanica* × (((*S. javanica* × *S. nigra*) × *S. nigra*) × *S*. *racemosa* − *miquelii*)	JA × (((JA × NI) × NI) × MIQ)	3
(*S javanica* × (*S. javanica* × *S. racemosa* − *miquelii*)) × ((*S. javanica* × *S. nigra*) × *S. nigra* ‘Black Beauty’)	(JA × (JA × MIQ)) × ((JA × NI) × BB)	1
((*S. javanica* × *S. nigra*) × *S. nigra* ‘Black Beauty’) × (*S. javanica* × *S. racemosa* − *miquelii*)	((JA × NI) × BB) × (JA × MIQ)	1
((*S. javanica* × *S. nigra*) × *S. racemosa* subsp. *sibirica*) × (*S. javanica* × *S. nigra*)	((JA × NI) × SIB) × (JA × NI)	1
Interspecific hybrids exhibiting similarity to *S. cerulea*		
*S. javanica* × *S. cerulea* No 3 C1	JA × CER No 3 C1	5
*S. javanica* × (*S. javanica* × *S. cerulea*)	JA × (JA × CER)	2
((*S. cerulea* × *S. nigra*) × *S. javanica*) × (*S. cerulea* × *S. nigra*)	((CER × NI) × JA) × (CER × NI)	1
Interspecific hybrids exhibiting combination of *S. cerulea* and *S. racemosa*		
(*S. javanica* × *S. racemosa*) × (*S. cerulea* × *S. nigra*)	(JA × RAC) × (CER × NI)	1
((*S. javanica* × *S. nigra*) × *S. racemosa* − *miquelii*) × (*S. cerulea* × *S. racemosa* − *miquelii*)	((JA × NI) × MIQ) × (CER × MIQ)	1
((*S. javanica* × *S. cerulea*) × *S. racemosa* − *miquelii*) × ((*S. javanica* × *S. nigra*) × *S. cerulea*)	((JA × CER) × MIQ) × ((JA × NI) × CER)	2
((*S. javanica* × *S. nigra*) × *S. racemosa* subsp. *sibirica*) × *S. cerulea*	((JA × NI) × SIB) × CER	1
((*S. javanica* × *S. nigra*) × *S. racemosa* subsp. *sibirica*) × (*S. javanica* × *S. cerulea*) No 3	((JA × NI) × SIB) × (JA × CER) No 3	5

The names *S. nigra*, *S. cerulea* and *S. racemosa* correspond to the names *S. nigra* subsp. *nigra*, *S. nigra* subsp. *cerulea* and *S. racemosa* subsp. *racemosa*, respectively, in the revised classification of Bolli [23]. *S. racemosa* subsp. *racemosa* also includes the taxa named as ‘*miquelii*’. ^a,b^—different individuals/genotypes from the same cross, C1 means first clonal generation. No.—number of sampled plants.

## Data Availability

Data are available in a publicly accessible repository.

## Supplementary Materials

The following are available online at https://www.mdpi.com/article/10.3390/plants10040653/s1, Figure S1: Variable correlation and scores plot of the first two principal components. Ellipses of clusters represent 0.68 normal probability level, Figure S2: Variable correlation and scores plot of the first and third principal component. Ellipses of clusters represent 0.68 normal probability level, Figure S3: Variable correlation and scores plot of the first and the fourth principal component. Ellipses of clusters represent 0.68 normal probability level.
