# Supplementary material for: The Distribution of Minerals in Crucial Plant Parts of Various Elderberry (Sambucus spp.) Interspecific Hybrids
Source: Plants (Basel). 2021 Mar 30;10(4):653. doi: 10.3390/plants10040653 (PMC8065488; doi:10.3390/plants10040653)
Supplement: Supplementary file 1 [file plants-10-00653-s001.pdf]

Supplemental material

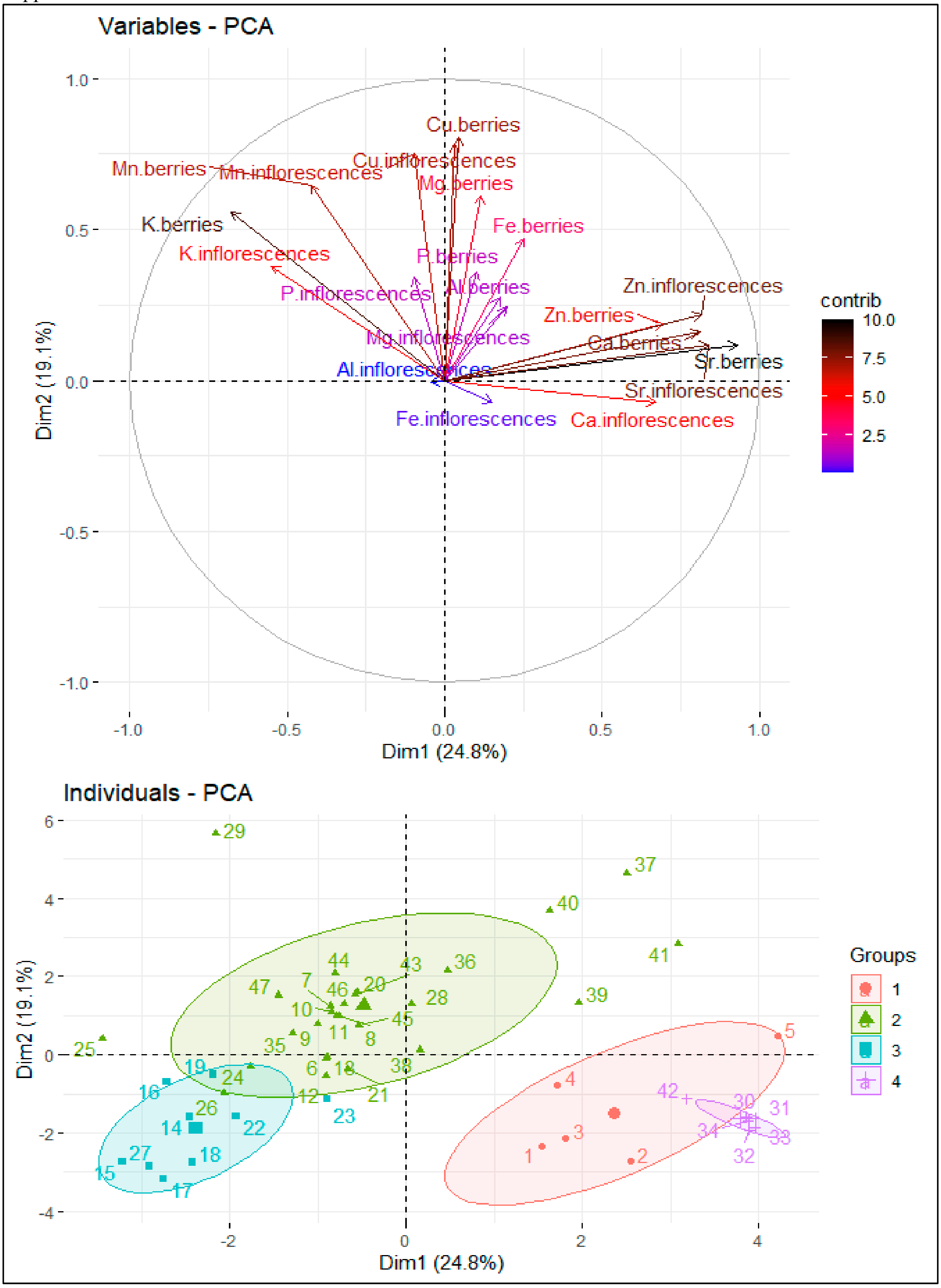

Supplemental material – Figure 1. Variable correlation and scores plot of the first two principal components. Ellipses of clusters represent 0.68 normal probability level.

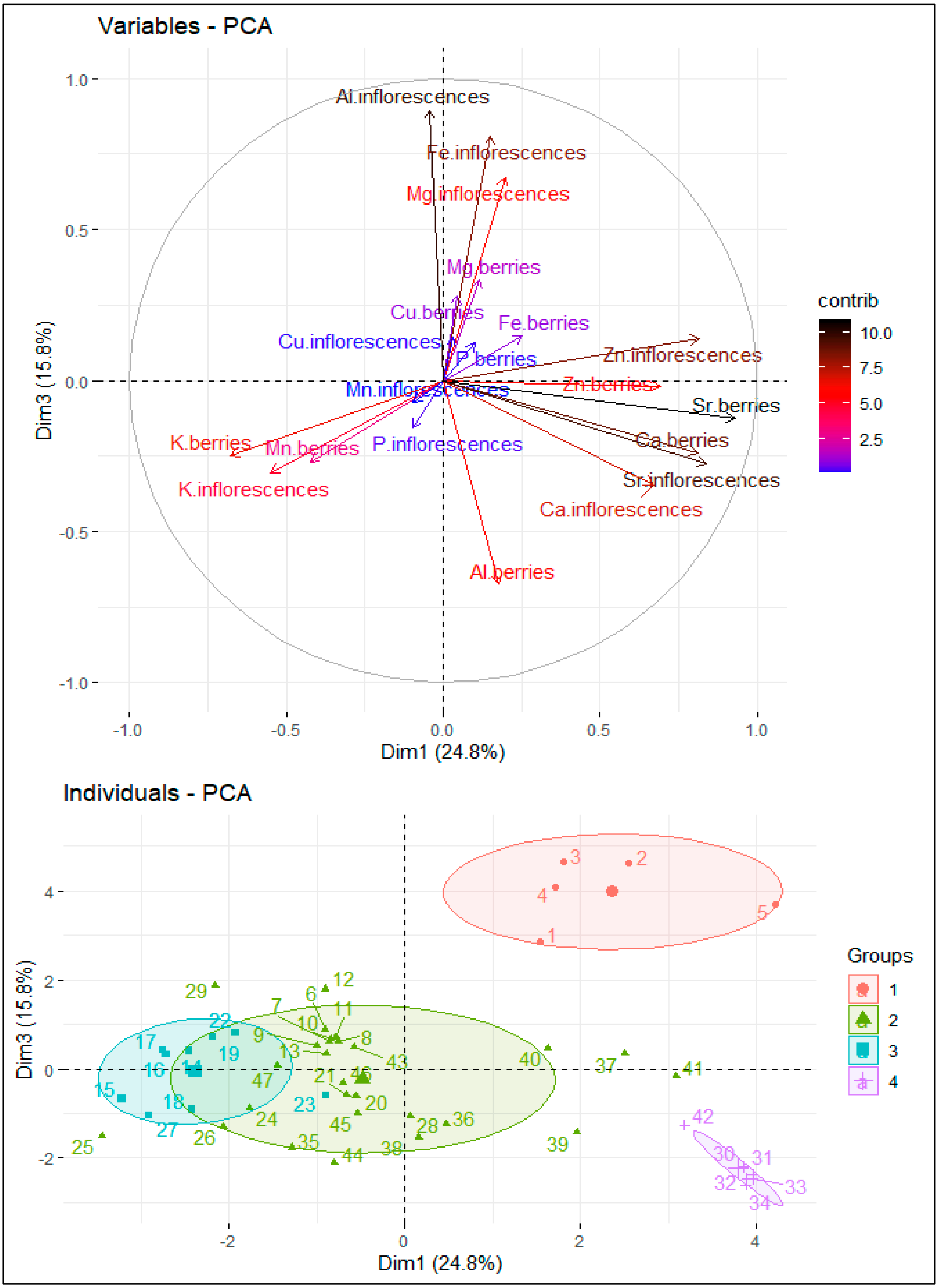

Supplemental material – Figure 2. Variable correlation and scores plot of the first and third principal component. Ellipses of clusters represent 0.68 normal probability level.

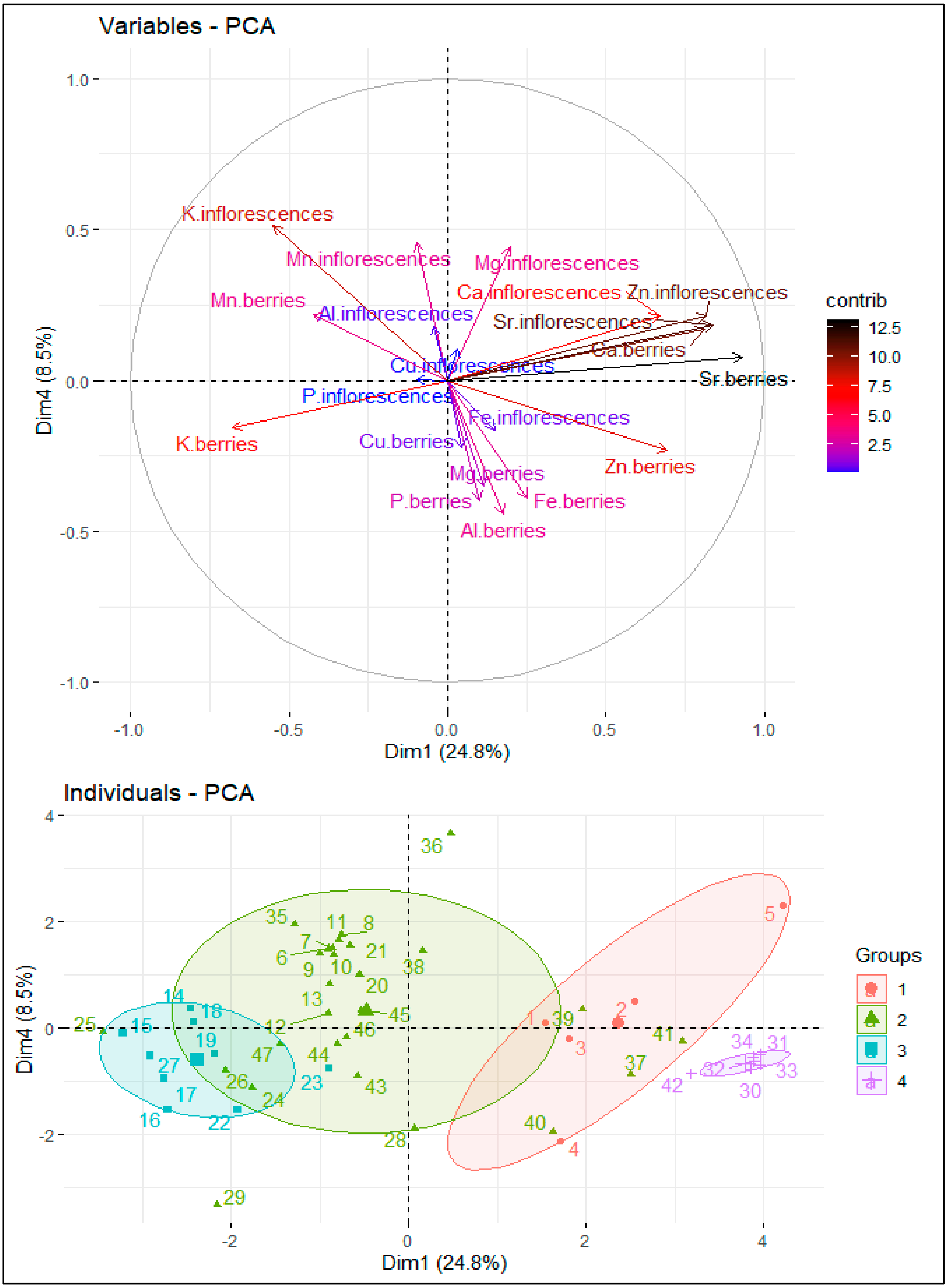

Supplemental material – Figure 3. Variable correlation and scores plot of the first and the fourth principal component. Ellipses of clusters represent 0.68 normal probability level.
